# Medium term outcome of Lancaster cortical window technique for extraction of femoral stem in revision hip arthroplasty

**DOI:** 10.1186/s13018-021-02458-7

**Published:** 2021-05-17

**Authors:** Amit Singh, Sunirmal Mukherjee, Kuntal Patel, Deepak Herlekar, Srikant Gandavaram, Nicholas Charalambous

**Affiliations:** University Hospital of Morecambe Bay NHS Foundation Trust, Ashton Road, Lancaster, LA1 4RP UK

**Keywords:** Revision hip arthroplasty, Extraction of the femoral stem, Osteotomy

## Abstract

**Background:**

The extraction of a femoral stem during the revision hip arthroplasty can be a daunting task and can lead to catastrophic complications for the patient. A sound technique employed intraoperatively helps in the speedy recovery of the patient and reduces the risk of future surgical interventions. In this study, we present a medium-term outcome of our novel Lancaster cortical window technique which can be used for the removal of cemented or uncemented femoral stems.

**Methods:**

The study was conducted at a specialist centre in the north-west of the UK from January 2014 to May 2019. This is a retrospective case series where patients were treated surgically using the Lancaster cortical window technique for removal of the femoral implant during a revision hip arthroplasty. Patient’s electronic notes and radiographs were used to evaluate the functional and radiological outcome.

**Results:**

In this study, 18 patients were managed surgically using the novel Lancaster window technique. The mean age of all the patients was 81.5 years, and the male to female ratio was 10:8. Fifteen patients underwent revision surgery for aseptic loosening of the femoral and acetabular components. The rest of the three patients had revision surgery for a broken femoral stem, intraoperative femoral canal perforation while implanting a total hip replacement femoral stem and infection. Twelve femurs were replanted with uncemented long femoral stems and six with long cemented stems. The cortical window osteotomy united in all the patients in 4.2 months (mean). The mean follow-up of these patients is 20.9 months, and none of them had any implant subsidence or loosening at the time of their last follow-up.

**Conclusion:**

We believe Lancaster cortical window technique can be safely used for the removal of cemented stems during revision hip arthroplasty without the need for expensive equipment.

## Introduction

Revision hip arthroplasty is a complicated procedure and is associated with significant risks for the patients [3]. A poor technique for extraction of components of a hip replacement can lead to complications such as fractures, massive blood loss, prolong surgical time and increased risk of post-operative infection [1]. The operating surgeon must be aware of the surgical techniques which can mitigate these risks.

A cemented femoral stem is implanted using a bone cement which acts as a grout instead of a glue. The bone cement forms a close interlock with the femoral stem and the bone [19]. Hence, extraction of this stem would require the removal of metalwork as well as the bone cement within the intramedullary canal [5]. The indications for removal of components include aseptic loosening, broken stem, infection and periprosthetic fractures [9].

The commonly used method for extraction of cemented femoral stem is extended trochanteric osteotomy [8]. But the technique is associated with a high risk of non-union and bone resorption [6]. We previously have described a novel cortical window technique at the lateral aspect of the femoral shaft for removal of the cemented femoral stem [17]. We now present detailed information on the Lancaster cortical window technique along with the medium-term functional and radiological outcome of our patients who underwent revision hip arthroplasty using this technique at our institute.

## Material and methods

This is a retrospective case series in which experienced revision arthroplasty surgeons operated on all the patients using the Lancaster cortical window technique as part of revision hip arthroplasty for the removal of the cemented femoral stem. The study was conducted at our institute in the north-west of the UK between January 2014 to May 2019. We included all the patients in this study who were managed surgically using the Lancaster cortical window technique. Data collection was performed using patient electronic notes and radiographs. The purpose of the study was to find out if our technique can be safely used for the removal of cemented femoral stem without increasing the risk of non-union, periprosthetic fracture and implant loosening. We also analysed the data to see if the patient’s immediate post-operative mobility was affected directly due to the cortical window. The mobility status and pain levels were assessed at regular follow-up, which was at 6 weeks, 3 months, 1 year and then yearly. All the patients included in this study remain under yearly or patient-initiated follow-up at our trust. The bony union of the cortical window was assessed on serial follow-up radiographs, and osteotomy was considered united when cortical bridging was noticed on anterior-posterior and lateral radiographs. Similarly, to assess implant subsidence, measurements were taken from the highest point of the greater trochanter (tip of the greater trochanter) to the highest point of the lateral aspect of the femoral stem as described in the article by Selvaratnam, Shetty and Sahni [18].

Below is the detailed description of the surgical technique along with the intraoperative images, which we hope will provide in-depth insight to the reader about this novel technique.

### Surgical technique

**Fig. 1:**
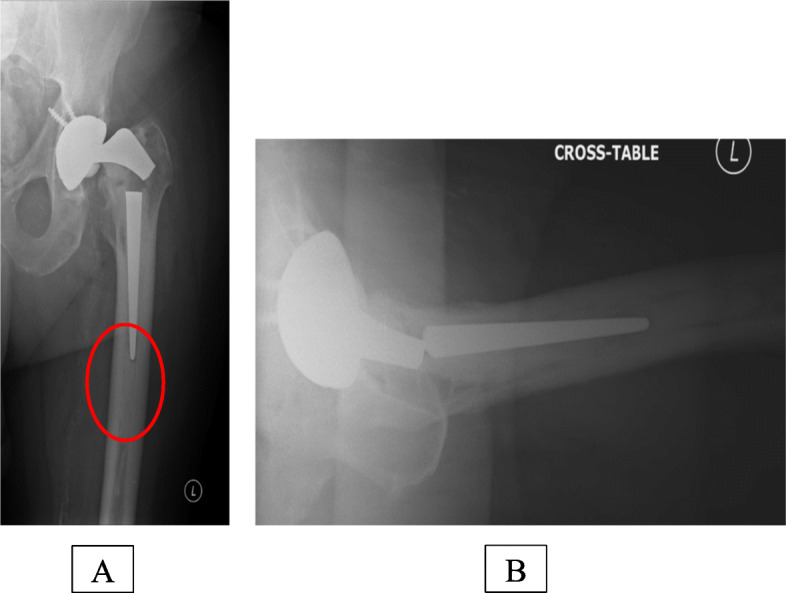
**a**, **b** Radiograph showing broken femoral stem. Note the cement column beyond the tip of the femoral stem in figure **a**

**Fig. 2:**
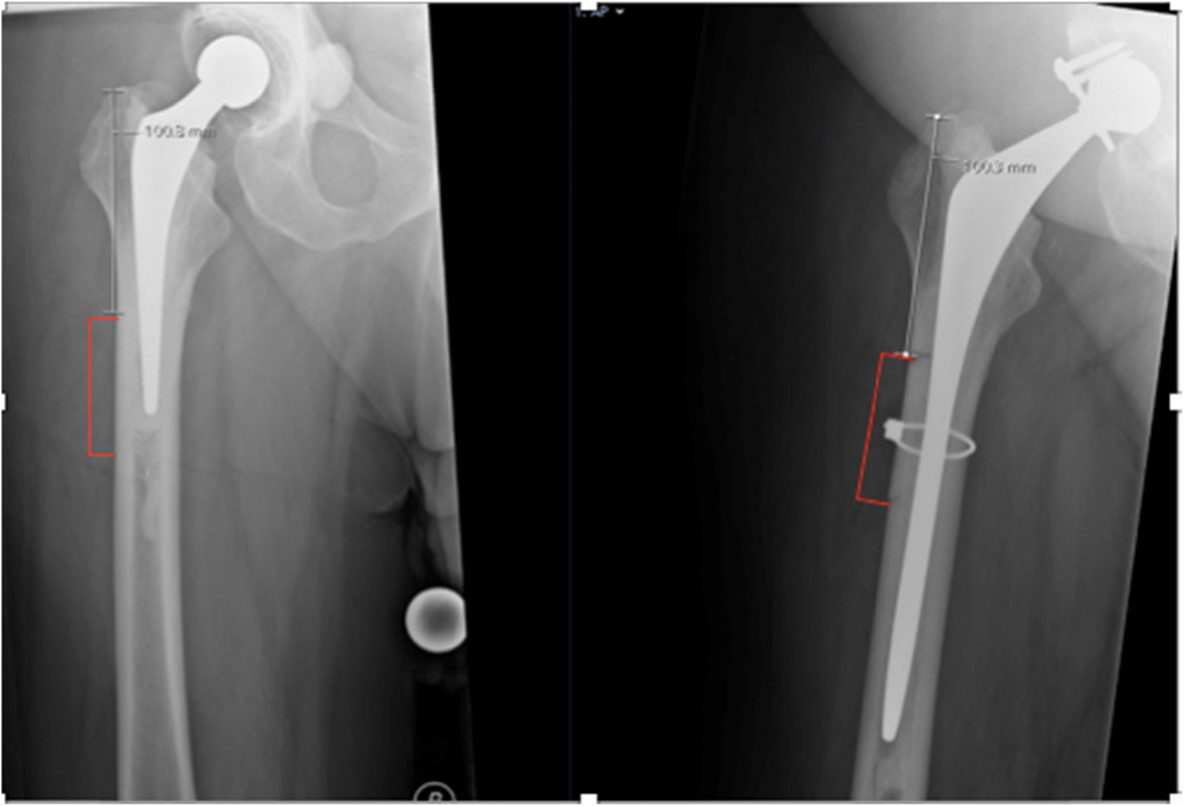
Radiographs of a patient with infected cemented THR, marked preoperatively for osteotomy site. Note the starting point of osteotomy site measured from the tip of the greater trochanter

The site for the cortical window should be marked preoperatively using radiographs. First, the starting point of the cortical window is measured from the tip of the greater trochanter. Following that approximate length of the osteotomy is marked to cover the end of the femoral stem, cement column beyond the tip of the stem and the cement restrictor (Figs. 1 and 2).

**Fig. 3:**
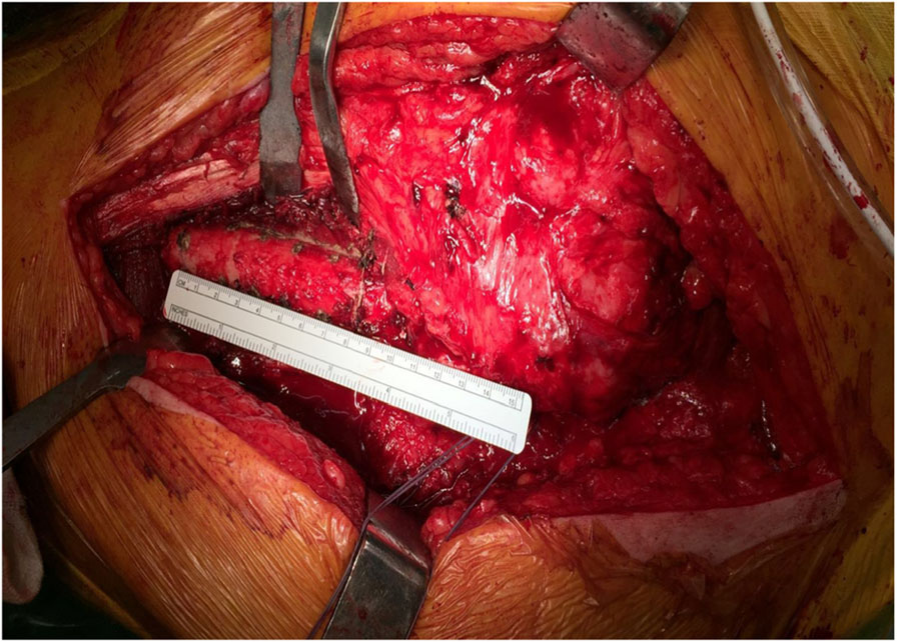
Intraoperative marking at the start and the end points of osteotomy

Intraoperatively, the vastus lateralis is split to expose the lateral aspect of the femoral shaft, and the starting point is marked at a prefixed distance from the tip of the greater trochanter using a sterile ruler (Fig. 3). The operating surgeon should aim to preserve as much soft tissue as possible to maintain a good blood supply to the bone. Occasionally, the femoral stem is loose and easily extracted after clearing the bone cement from the shoulder of the implant. However, osteotomy may still be required to clear the cement column beyond the tip of the femoral stem as shown in Fig. 1a, b. In this situation, the femoral stem removed can itself be used as a template to mark the osteotomy site by placing the stem along the lateral aspect of the femoral shaft. The average width of the osteotomy is 2.5 cm, but it also depends upon the width of the lateral cortical surface.

**Fig. 4:**
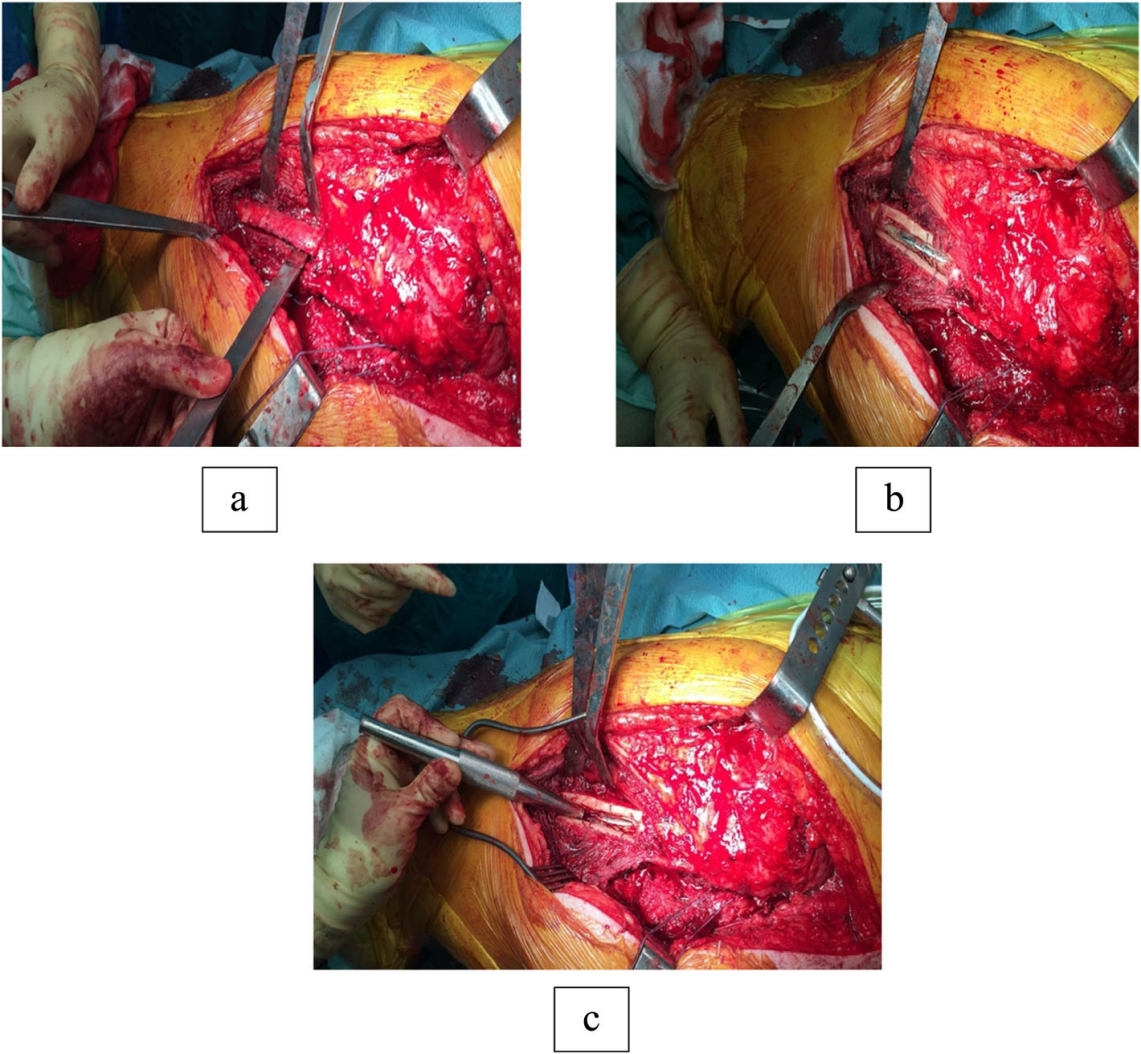
**a** Creating a window using a narrow osteotome. **b** Lancaster cortical window along the lateral femoral cortex with exposed cement and femoral stem**. c** Retrograde extraction of the stem once the bone cement is cleared around the femoral stem

Now the four corners of osteotomy are drilled with a 2-mm drill bit. A narrow oscillating saw is used to connect these four corners, and the osteotomy is completed with an osteotome. The saw and the osteotome should be angled to about 45° to create a sloping edge which increases the surface area and improves healing as shown in Fig. 4a The window is a rectangular shape, and any sharp corners should be avoided to reduce the risk of a stress riser. The cortical window can now be used to assess the femoral stem, the cement and the cement restrictor (Fig. 4b). A cerclage wire can also be placed in the beginning below the site of the cortical window to prevent any fractures. It is particularly important in patients with significant bone loss due to osteolysis.

**Fig. 5:**
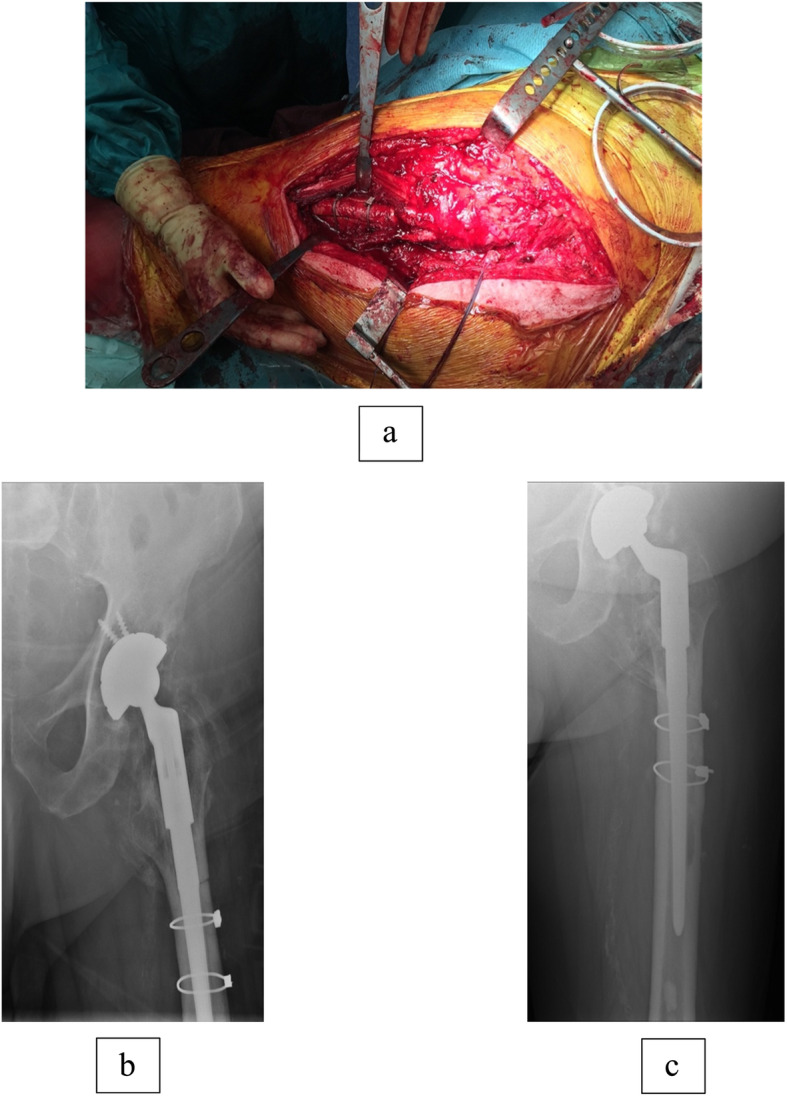
**a** Intraoperative image of stabilization of window with cerclage wires. **b** Immediate post-operative radiographs. **c** Two years follow-up radiograph with healed cortical window osteotomy

After removing the femoral stem and bone cement from the intramedullary canal, the square piece of bone is replaced, and the window is stabilised with one or two cerclage wires (Fig. 5a). If there are concerns about bone healing, one can also consider using bone grafting. The surgeon can now proceed with the preparation of the femoral canal for reimplantation of cemented or uncemented femoral stem.

## Results

From January 2014 to May 2019, 18 patients were surgically managed using the Lancaster cortical window technique at our specialist centre. The mean age of these patients was 81.5 years (range 76–94 years), and the male to female ratio was almost equal (M: F = 10:8). Two patients in this study had a background of inflammatory arthritis, but this did not adversely affect the outcome.

The indications for revision hip arthroplasty for all the patients have been described in Table 1. One patient had a perforation of the femoral canal while undergoing revision arthroplasty for aseptic loosening of a total hip replacement. This intra-operative complication was recognised after the implantation of the cemented femoral stem. Hence, the patient underwent removal of the cemented femoral stem by our team using the cortical window technique (Table 1).

**Table 1:** Indications for revision arthroplasty and patient distribution

Indication	Number of patients
Aseptic loosening	15
Infection	1
Broken femoral stem	1
Intraoperative femoral canal perforation	1

All the revision surgeries were performed using the posterior approach. In all the patients, both acetabular cup and femoral stem were revised except in the patient with the broken femoral stem of the primary hip replacement (Table 2). Twelve patients received long uncemented femoral stem where primary stability is achieved by diaphyseal fixation, and the rest of the six patients underwent reimplantation with a cemented femoral stem. The cortical window in all the patients was stabilised using one or two cerclage wires. No bone graft was used in any of the patients (Fig. 5).

**Table 2:** Distribution of patients as components revised, technique of revised femoral stem implantation and cortical window union rate

Surgical intervention	No. of patients
Acetabular cup revised	17
Femoral stem revised	18
Revision with long uncemented stem	12
Revision with long cemented stem	6
Cortical window union	18

The cortical window radiologically healed by 4.2 months (mean) in all the patients except two who did not have a regular follow-up after the initial visit to the clinic at 6 weeks. These two patients were medically unwell from causes unrelated to the surgery. However, these two patients did have a yearly follow-up, and at their latest visit to the hospital, the radiographs conformed that osteotomies have healed. In the immediate post-operative period, 16 out of 18 patients were able to mobilise bearing full weight over the operative limb. Two patients were advised to maintain protected weight-bearing for 6 weeks as there was significant bone loss around the femoral stem due to osteolysis. Both the patients received long uncemented femoral stem.

The mean follow-up in this study is of 20.9 months. At the most recent follow-up of 18 patients, all of them were mobilising pain-free, bearing full weight over the operated limb. Eight out of eighteen patients needed a single stick for support due to factors such as advancing age or worsening arthritis in the ipsilateral knee (Table 3). No change was noticed in the femoral stem position in the form of subsidence or implant loosening.

**Table 3:** Mobility and pain status of patients at the last follow-up

Mobility status	No. of patients	Pain
Full weight bearing	10	No
Full weight bearing with support	8	No

We had three patients who had hip dislocation within 3 months of the surgery. Out of three, two patients needed further surgical intervention in the form of an application of constrained acetabular liner and posterior lip augmentation device. One patient had closed reduction of the hip, and as the hip was stable, no further surgical intervention was required. This patient did not have any additional episodes of hip dislocation during the follow-up. None of the patients had any intraoperative fractures. But two patients had distal femur fracture after 3 months of revision surgery and needed internal fixation with locking plate and screws. The fractures were distant from the cortical window site, and osteotomy has healed at the time of these injuries.

## Discussion

Total hip replacement is considered one of the most successful orthopaedic procedures and has a high patient satisfaction rate [10]. As per the UK National Joint Registry, the 15-year revision rate for a cemented hip replacement is 5.46% [16]. At the time of revision hip replacement surgery, removal of the components of a cemented hip replacement requires specialised skills and instruments [8]. The most common surgical technique described in the literature is extended trochanteric osteotomy (ETO) [11]. The ETO can also be used to correct varus femoral deformity, which is secondary to bone remodelling around a loose femoral component [20]. But the current literature suggests that ETO is also associated with complications such as arterial injury, intra- and post-operative fractures, proximal migration of osteotomy fragment and non-union [21]. The non-union of the osteotomy can lead to the functionless greater trochanter and hip abductors which can lead to limping gait and chronic pain for the patients [2]. One of the advantages of our technique is the low risk of non-union and nil effect on hip abductor mechanism.

There have been several versions of cortical osteotomy described in the literature for the extraction of the broken femoral stem [1] or removal of the cemented/uncemented femoral stem [7]. But these are the technique described in a case report or in the form of a case series with smaller sample size and limited follow-up. This affects the external validity of these studies. There is also a lack of insight into the long-term consequences of these techniques, such as implant subsidence or loosening. Park et al. [15] described their version of the anterior cortical window technique for the removal of the femoral stem during revision arthroplasty. In this study, the implant subsidence rate was 8.4% (within 1 year of surgery), and the non-union rate for the cortical window was 2%. The reoperation rate was also significantly high, i.e. 21.4% due to factors such as loosening of the femoral stem and acetabular cup, bursitis related to cerclage wire, periprosthetic femur fracture, prosthetic joint infection and superficial wound infection. The technique is also different from ours as the length of the window is almost equal to an ETO, extending from the shoulder of the stem to the end of the cement restrictor. This technique does warrant patients mobilise with protective weight bearing over the operated limb for 6 weeks.

In our study, all cortical windows united mainly due to the smaller size of the window in addition to the meticulous surgical technique. The bevelled edge created by angulating the saw while making the window increases the surface area, and once the window is reduced, it heals with primary healing [12]. The osteotomy was considered healed when cortical bridging was noticed on anterior-posterior and lateral radiographs [4]. We did not see a change in the implant position in any of our patients during the follow-up, and all the patients were allowed full weight bearing over the revised hips.

Melmer et al. [13] in their study described a long anterolateral cortical window which is made near the tip of the stem. Unfortunately, the author fails to explain the methodology of deciding the site of the window. A window made far from the implant tip will require alteration and further extension. This can potentially increase the length of the window and also the associated risks such as periprosthetic fracture and implant subsidence. In their study, 5.8% of the patients had implant subsidence post-osteotomy. To avoid such complications, we believe that it is crucial to mark the osteotomy site by assessing the preoperative radiographs or CT scans as described in our surgical technique.

We acknowledge that there are limitations to this study. This is a retrospective case series and provides level IV evidence [14]. But the follow-up of almost 2 years of the majority of the patients in this study provide a strong evidence that this technique does not increase the risk of intraoperative fractures and implant failure.

## Conclusion

We believe the surgical technique described in this study is reproducible and has fewer complications. The procedure does not warrant any unique instrument and hence avoids the need for surgeons to add a more complex and expensive kit to their standard revision instrument set. Due to the compact size of the osteotomy and inherent stability, patients can be allowed to full weight bear in the immediate post-operative period, and this expedites patient rehabilitation and in turn shorter inpatient stay.

## Data Availability

The datasets used and analysed during the current study are available from the corresponding author on reasonable request.

## References

[1] Akrawi H, Magra M, Shetty A, Ng A (2014). A modified technique to extract fractured femoral stem in revision total hip arthroplasty: a report of two cases. Int J Surg Case Rep..

[2] Brun O, Maansson L (2013). Fractures of the greater trochanter following total hip replacement. HIP Int.

[3] Dy C, Bozic K, Pan T, Wright T, Padgett D, Lyman S (2014). Risk factors for early revision after total hip arthroplasty. Arthritis Care Res.

[4] Fisher J, Kazam J, Fufa D, Bartolotta R (2018). Radiologic evaluation of fracture healing. Skeletal Radiol..

[5] Hofmann A, Anderson M (1987). Removal of cemented total hip components. Tech Orthop.

[6] Hellman E, Capello W, Feinberg J (1998). Nonunion of extended trochanteric osteotomies in impaction grafting femoral revisions. J Arthroplasty..

[7] Harada H, Fujita H. A novel antero-medial cortical window technique for removal of well-fixed fully porous stem in revision total hip arthroplasty. Orthop Muscular Syst. 2016;05(02):3-4.

[8] Kraay MJ (2015). Cemented component removal: tricks of the trade. Orthopaedic Proceedings.

[9] Laffosse J (2016). Removal of well-fixed fixed femoral stems. Orthop Traumatol Surg Res.

[10] Learmonth I, Young C, Rorabeck C (2007). The operation of the century: total hip replacement. Lancet..

[11] Masri B, Mitchell P, Duncan C (2005). Removal of solidly fixed implants during revision hip and knee arthroplasty. J Am Acad Orthop Surg.

[12] Marsell R, Einhorn T (2011). The biology of fracture healing. Injury..

[13] Melmer T, Steindl M, Schiessel A, Zweymüller KA (2004). Fenestration of the femoral shaft: a standard procedure in revision hip surgery without bypassing the cortical defect. Orthopedics..

[14] Murad M, Sultan S, Haffar S, Bazerbachi F (2018). Methodological quality and synthesis of case series and case reports. BMJ Evid Based Med.

[15] Park C, Yeom J, Park J, Won S, Lee Y, Koo K (2019). Anterior cortical window technique instead of extended trochanteric osteotomy in revision total hip arthroplasty: a minimum 10-year follow-up. Clin Orthop Surg.

[16] Reports.njrcentre.org.uk [online]. Available from: https://reports.njrcentre.org.uk/Portals/0/PDFdownloads/NJR%2016th%20Annual%20Report%202019.pdf. Accessed 13 May 2020.

[17] Singhai S, Gandavaram S, Herlekar D, Patel K. Effectiveness of cortical window technique for revision hip arthroplasty. Orthop J MP Chapter. 2019;25(1).

[18] Selvaratnam V, Shetty V, Sahni V (2015). Subsidence in collarless Corail hip replacement. Open Orthop J.

[19] Vaishya R, Chauhan M, Vaish A (2013). Bone cement. J Clin Orthop Trauma..

[20] Valle C, Berger R, Rosenberg A, Jacobs J, Sheinkop M, Paprosky W (2003). Extended trochanteric osteotomy in complex primary total hip arthroplasty. J Bone Joint Surg Am.

[21] Wronka K, Gerard-Wilson M, Peel E, Rolfson O, Cnudde P (2020). Extended trochanteric osteotomy: improving the access and reducing the risk in revision THA. EFORT Open Rev.

